# A Peculiar Case of Nymphomania*Read before the I. H. A. June, 1884.

**Published:** 1884-10

**Authors:** E. W. Berridge

**Affiliations:** London


### A PECULIAR CASE OF NYMPHOMANIA.*

E. W. Berridge, M. D., London.

December 29th, 1881, Mrs. ——, æt. forty-one, consulted me for the following condition: Since childhood, as long as she can remember (and she can remember back to the age of three), any annoyance or hurry will bring on intense sexual pleasure, extending from sexual organs all over body. This will never culminate of itself, except during sleep; but she has to complete the orgasm by moving violently about as she sits on a chair. If she does not thus voluntarily terminate it, the desire will last off and on the whole day, passing off during sleep. It passes off with discharge from vagina, as in coition. When it occurs during sleep it usually wakes her. The excitement during sleep is always caused by dreams of hurry and annoyance.

She was married at age of twenty-one and left a widow at age of twenty-eight. Has had no children, but has had three miscarriages—one at third month and two at seventh week. She never had any sexual pleasure, unless very slightly, during

---

* Read before the I. H. A. June, 1884.

coition; never had any natural sexual desire for men; but this abnormal excitement continued during her married life and ever since without varying much. Menses appeared at age of seventeen—never regular—either too soon or too late. Menopause not yet arrived; great dysmenorrhœa before marriage—less since; menses scanty—*never* more than three days in duration—dark, *always* clotted, generally at intervals of twenty-four days—sometimes of five weeks. At age of eighteen or nineteen had weak spine; could walk easily, but could not rise from the recumbent posture without help. She was treated by a professed homœopathic physician, who ordered cold hip-baths for twenty minutes each day in addition to medicinal treatment. She recovered slowly, being ill for months. After the spinal illness her sight failed, continuing bad ever since, though she has consulted many allopaths and pseudo-homœopaths in vain. For from two to four years she was able to restrain herself from the completion of the orgasm, except during sleep, till one day last summer she failed to do so, after which occurrence she felt a sharp prick like a pin in left eye, and at times since; the sight has been worse since then. The sight is now dim, as from a haze, especially in left eye; left pupil does not react well to light. Since the spinal disease the right eye has turned in, especially noticeable if she is excited, worried, or tired. Two years ago she had fetid leucorrhœa, which a professed homœopath suppressed with a medicated injection. For some months has had occasionally a violent pain just within anus, nearer the left side than the right, followed, as the pain abated, by a feeling of dying, accompanied by ghastly appearance of face. The pain lasts about ten minutes. Constipated all her life; no stool for a week unless she takes a purgative, and then the stools are too large and hard, passing with great straining pains, as if anus were being torn open. An enema brings nothing away. Has taken *Tamar Indien* for months. At times she has been quite free from constipation, but it has been much worse for the last six months. Is excessively fond of little children.

The sexual symptoms so much resembled those described in Fincke's proving of *Lachesis*, recorded in *Hahnemannian Monthly*, Vol. I, but omitted in Allen's *Encyclopædia*, that I decided on that remedy, and gave her a daily dose, for seven days, of *Lachesis*$^{cm}$ (F. C.).

Jan. 13th, 1882.—Reports that she has only taken five doses of *Lachesis*. She took it regularly for the first three days, then, finding that the bowels did not act and that giddiness came on, as it always does when constipated, she took a dose of *Tamar*

*Indien*, then after a day's interval took two more doses of *Lachesis*, since which time she has left it off and taken a daily dose of *Tamar Indien*, but still remains constipated. The sexual excitement is certainly better. It has only occurred once, and that was during sleep on January 1st, and it did not wake her. Previously had had heaviness of head, which this relieved. Her external conditions have remained unchanged. Menses have returned without any special pain, rather dark, but no clots; sight and eyes unchanged.

As the *Lachesis*—which seemed fairly indicated by the rectal symptoms and strongly indicated by the sexual symptoms—had failed to relieve the constipation, I concluded that there was some opposing cause to be found in her mode of living. On inquiry, I found that she drank much strong tea and took scarcely any exercise. I gave no more medicine, but ordered her to take more exercise, less tea, and to substitute for it the *Pure Solidified Cocoa*.*

Jan. 25th.—Has taken the Cocoa once daily, except yesterday; less tea and more exercise. Constipation has not returned and she has taken no more purgatives; no return of sexual excitement; the occasional violent pains just within anus have not returned since first consultation; sight a little clearer, pupil acts better; right eye still turns in.

Feb. 1st.—No more constipation; sight about the same; very slight sexual symptoms three times since last visit.

Feb. 10th.—No constipation; has only twice had the sexual excitement, and then very slightly so—even slighter than before; no more pricking in eye; sight a little better.

May 5th.—Reports that about the early part of March she had the sexual excitement in sleep for three consecutive nights—the first night three times, the others only once; has not had any to speak of at any other time; soon afterward the sight of left eye began to get worse again; once since her last consultation worry seemed to bring on the constipation, for which she took a dose of *Tamar Indien*, but has had no constipation at any other time; eyes have remained otherwise unchanged; feels generally well and much stronger, though she has been much troubled about the serious illness of a relative.

As the action of the medicine now seemed quite exhausted, I gave her *Lachesis*$^{5mm}$ (Fincke) every other day for fourteen days.

---

* NOTE.—The only preparation with which I am acquainted which is both *perfectly* pure and contains *all* the nutritive ingredients of the Cocoa. Sold by Alfred Heath & Co., 114 Ebury St., S. W.

May 21st.—Reports sight some improved, and for the last three or four days there has been a still further improvement; the sexual symptoms scarcely trouble her at all.

Sept. 26th.—Reports that her relative died at end of May, and ever since has had at times feeling as if she were going to die, but without faintness; it begins in epigastrium and spreads all over her, lasting from a few minutes to an hour, followed by weakness for the rest of the day. During the attack looks ghastly; the attacks occur from two or three times a week to once a fortnight; has very seldom had any sexual excitement since last consultation, and never to any uncontrollable degree. Since the loss of her relative she has again been troubled with constipation. I ordered her to resume the Cocoa, which she had omitted for some time, and to take more exercise. I also prescribed *Phosphorus*$^{cm}$ (F. C.) every other day for fourteen days.

Jan. 12th, 1883.—Wrote to say that the medicine had been "quite successful."

